# Delimitation of the *Earliness per se D1* (*Eps-D1*) flowering gene to a subtelomeric chromosomal deletion in bread wheat (*Triticum aestivum*)

**DOI:** 10.1093/jxb/erv458

**Published:** 2015-10-17

**Authors:** Meluleki Zikhali, Luzie U. Wingen, Simon Griffiths

**Affiliations:** ^1^John Innes Centre, Norwich Research Park, Norwich, Norfolk, UK.

**Keywords:** *Arabidopsis thaliana*, *Brachypodium distachyon*, *Eps-D1*, flowering, *Triticum aestivum*, *Triticum monococcum*, wheat.

## Abstract

Earliness *per se* (*Eps*) genes account for the variation in flowering time when vernalization and photoperiod requirements are satisfied. Genomics and bioinformatics approaches were used to describe allelic variation for 40 *Triticum aestivum* genes predicted, by synteny with *Brachypodium distachyon,* to be in the 1DL *Eps* region. Re-sequencing 1DL genes revealed that varieties carrying early heading alleles at this locus, Spark and Cadenza, carry a subtelomeric deletion including several genes. The equivalent region in Rialto and Avalon is intact. A bimodal distribution in the segregating Spark X Rialto single seed descent (SSD) populations enabled the 1DL QTL to be defined as a discrete Mendelian factor, which we named *Eps-D1*. Near isogenic lines (NILs) and NIL derived key recombinants between markers flanking *Eps-D1* suggest that the 1DL deletion contains the gene(s) underlying *Eps-D1*. The deletion spans the equivalent of the *Triticum monoccocum Eps*-*A* ^*m*^ *1* locus, and hence includes *MODIFIER OF TRANSCRIPTION 1* (*MOT1*) and *FTSH PROTEASE 4* (*FTSH4*), the candidates for *Eps*-*A* ^*m*^ *1*. The deletion also contains *T. aestivum EARLY FLOWERING 3-D1* (*TaELF3-D1*) a homologue of the *Arabidopsis thaliana* circadian clock gene *EARLY FLOWERING 3*. *Eps-D1* is possibly a homologue of *Eps-B1* on chromosome 1BL. NILs carrying the *Eps-D1* deletion have significantly reduced total *TaELF3* expression and altered *TaGIGANTEA* (*TaGI*) expression compared with wild type. Altered *TaGI* expression is consistent with an *ELF3* mutant, hence we propose *TaELF3-D1* as the more likely candidate for *Eps-D1*. This is the first direct fine mapping of *Eps* effect in bread wheat.

## Highlight

The major flowering time genes cloned to date regulate photoperiod and vernalization response. We identified a deletion containing genes regulating earliness *per se*, which fine tune flowering in hexaploid wheat.

## Introduction

Humans consume most of their calories from wheat and 20% of the dietary protein for developing countries is obtained from wheat (Reynolds *et al.*, 2012), which makes wheat strategic for global food security. Wheat is widely consumed across the globe because it can be grown in a wide range of environments (Slafer and Rawson, 1994). The major problem is that even though wheat yields are increasing, the yields are not keeping up with demand (Coff *et al.*, 2008; Gupta *et al.*, 2008; Lopes *et al.*, 2012). The challenge is to bridge the gap between wheat demand and wheat production (Dixon *et al.*, 2009; Rosegrant and Agcaoli, 2010). A better understanding of the mechanism of *Eps* leading to breeding by design for adaptive traits will be an important part of this process.

One of the important processes that controls grain number and quality, and hence yield, is the genetic and environmental control of the timing of flowering (Fjellheim *et al.*, 2014). Three major classes of genes control the timing of flowering. The photoperiod genes enable plants to perceive changes in day length while the vernalization genes enable response to exposure to extended periods of cold. In this way photoperiod and vernaliation genes control adaptation to mega environments. The third class is *Eps* genes, which control flowering time when both photoperiod and vernalization requirements are met (Slafer and Rawson, 1994), and act in fine tuning flowering time within mega environments (Zikhali and Griffiths, 2015).

The major photoperiod and vernalization genes of bread wheat have been positionally cloned (Yan *et al*., 2003, 2004, 2006; Turner *et al.*, 2005), but the identification of *Eps* genes has remained elusive in bread wheat. However, two Triticeae *Eps* loci have been cloned to date. One in *Triticum monococcum* on chromosome 3A (*Eps*-3*A* ^*m*^) for which the candidate gene is an orthologue of the *Arabidopsis thaliana LUX ARRHYTHMO*/ *PHYTOCLOCK 1* [(*LUX*/*PCL1*, which acts by disturbing the circadian clock (Shindo *et al.*, 2002; Gawrosnski and Schnurbusch, 2012; Mizuno *et al.*, 2012; Gawrosnski *et al*., 2014)]. The other is the *Hordeum vulgare EPS2* on chromosome 2H, which is orthologous with the wheat group 2 loci (Laurie *et al.*, 1995; Laurie, 1997). The candidate gene for EPS2 is a homologue of the *Antirrrhunum* gene *CENRORADIALIS* (*CEN*) designated *HvCEN* (Comadran *et al.*, 2012).

In barley, the *EARLY FLOWERING 3* gene (Boden *et al.*, 2014)—referred to also as *EARLY MATURITY 8* (*eam8*) (Faure *et al.*, 2012) or *Praematurum-a* (*Mat-a*) (Zakhrabekova *et al.*, 2012)—has been shown to play a major role in flowering time. Faure *et al.* (2012) and Zakhrabekova *et al.* (2012) both showed that mutants of this gene have an impaired circadian clock. The most recent report (Boden *et al.*, 2014) went further and showed that the wild type *EARLY FLOWERING 3* prevents flowering under short days by preventing gibberellin production and suppressing *FLOWERING LOCUS T1* (*FT1*) expression.

Further effort to clone *Eps* genes in *T. monococcum* include the thermo-sensitive earliness *per se* (*Eps*-*A* ^*m*^ *1*) locus (Bullrich *et al.*, 2002) for which two possible candidates—*MODIFIER OF TRANSCRIPTION 1* (*MOT1*) and *FTSH PROTEASE 4* (*FTSH4*)—have been suggested (Faricelli *et al.*, 2010). These studies are of particular relevance to the current study. The *T. aestivum* homologues of *MOT1* and *FTSH4* will hereafter be referred to as *TaMOT1* (generic) and *TaFTSH4* (generic) and *-A1*, *-B1* as well as *-D1* suffix will designate the three homoeologues, respectively.

The *Eps* effect is an important adaptive trait but the genes controlling it are not well understood. For example, the *Eps*-*A* ^*m*^ *1* and the *Eps*-3*A* ^*m*^ loci are both thermo-sensitive and determine the number of spikelets as well as the number of grains per spike, in addition to regulating flowering time (Bullrich *et al.*, 2002 Lewis *et al.*, 2008; Gawrosnski *et al*., 2014). One possible reason why *Eps* genes have not been well studied is that they were often mapped in crosses segregating for *Ppd and Vrn*, which often mask their effects.

Here we describe the high resolution mapping of an *Eps* quantitative trait locus (QTL) on the long arm of wheat chromosome 1 (1DL), which we named *Eps-D1* that was originally identified using doubled haploid populations (Griffiths *et al.*, 2009). We recently validated the 1DL QTL as an *Eps* effect using near isogenic lines (NILs) (Zikhali *et al.*, 2014) and now we show, using NILs and recombinants, that a chromosomal deletion which contains several genes including the wheat homologue of the *Arabidopsis* circadian clock gene *Early Flowering 3* (*ELF3*) is the likely cause of the *Eps-D1* effect.

## Materials and methods

### Plant growth: doubled haploid populations

Ninety-six lines each from three independent doubled haploid (DH) populations of crosses between Spark X Rialto, Avalon X Cadenza, and Malacca X Hereward, were grown in 1L pots. The growth conditions were as decribed for Spark X Rialto NILs in our earlier report (Zikhali *et al.*, 2014). For each of the 96 DH lines from the three populations, nine seeds were sown and germinated between 15–20 °C for 2 weeks. The nine seedlings of each line were then separated into three photoperiod treatments (each treatment had three plants of each line from the three DH populations). All treatments were sown on 21 December 2011 and germinated in a heated glasshouse up to 9 January 2012. The heating was then turned off to allow natural vernalization to commence on 10 January 2012 and heating was kept off up to 6 March 2012 to allow 8 weeks of vernalization. From 17 February 2012, all the benches with the three treatments were adjusted to move into the shed at 1700 hours (hrs) and out of the shed at 0700 hrs to give all treatments 10h of sunlight up to 6 March 2012 when vernalization ended. After the 8 weeks vernalization, one of the three treatments remained exposing plants to short days (SD, 10h light and 14h of darkness) by moving the plants out of the shed at 0700 hrs and then moving them back into the shed at 1700 hrs. The other two photoperiod treatments were adjusted to move out of the shed at 0600 hrs and back into the shed at 1800 hrs giving these bences 12h of natural light. Then additional lighing in the shed was used to give long days (LD, 16h light) and very long days (VLD, 20h light). Additional lighting was provided using 4h and 8h artificial white light using eight tungsten bulbs spaced 0.9 m apart delivering 1 μmol s^–1^ m^–1^ to augment the LD and VLD, respectively. The temperature was adjusted to 13–18 °C in glasshouse conditions using heaters. Heading date was measured on the leading tiller at Zadoks growth stage 55 (Zadoks *et al.*, 1974). The heading date scores were then used to carry out QTL confidence interval mapping (CIM) analysis using the R software (vs. 3.02) programme (R Core Team, 2013).

### Single seed descent populations

The single seed descent (SSD) populations were derived from a cross between Spark and Rialto. The same Spark X Rialto DH lines SR9 and SR23 used to develop the NILs in our earlier report (Zikhali *et al.*, 2014) were used to develop the SSD populations. The only difference between the NILs and SSD lines is that after screening the self fertilized heterozygotes from backcross 2 (BC_2_), the homozygotes at the same markers—*Xcfd63*, *Xgdm111*, and *Xbarc62*—were selected for the NILs (Zikhali *et al.*, 2014) while, for the SSD population, the heterozygotes at the three markers were selected for self-fertilization to form the recombinant SSD populations.

A population of 266 Backcross 2 F3 (BC_2_F_3_) individuals from four maintained independent populations was grown using SSD, up to BC_2_F_5_. Cross pollination was avoided in the four populations by covering spikes with cellophane bags before anthesis. When the SSD lines reached BC_2_F_5_, four plants were grown in randomized blocks for each SSD line in 1L pots and vernalized under short days (10h light) for 8 weeks at 6–10 °C in a controlled environment room. After the 8 weeks vernalization, the plants were then grown at 16h light with 4h supplementary light.

### Identification and design of additional molecular markers on 1DL

The *Xbarc62* marker (Song *et al.*, 2005) was the most distal on 1DL for the Spark X Rialto DH population and was at the peak of the 1DL QTL. We therefore determined the physical position of *Xbarc62* on 1DL in order to develop more markers to fine map the 1DL *Eps* effect. We retrieved from the National Centre for Biotechnology Information (NCBI) database the sequence BV211449 (Song *et al.*, 2005) used to develop the *Xbarc62* simple sequence repeat (SSR) marker. We then used the sequence to conduct homology searches (Altschul *et al.*, 1990) of the unassembled reads of the Chinese Spring 454 sequence database (Brenchley *et al.*, 2012). The BV211449 sequence matched the 3′ untranslated region (3′UTR) of the *T. aestivum Early Flowering 3* (*TaELF3*) (Higgins *et al.*, 2010) Genbank accession number AK332315 (Kawaura *et al.*, 2009). We also aligned the BV211449 sequence, which contains the ATCT repeat (*Xbarc62*) (Song *et al.*, 2005), with sequences from part of exon 4 and the 3′UTR of *Aegiolps tauschii ELF3* (*AtELF3*), *Triticum urartu ELF3* (*TuELF3*), and of the A, B, and D homoeologues of the Chinese Spring *TaELF3* gene.

We then used synteny between wheat and Brachypodium (Faricelli *et al.*, 2010; Higgins *et al.*, 2010; Faure *et al.*, 2012; Zakhrabekova *et al.*, 2012) to design 1DL specific markers on both the distal and proximal side of *Xbarc62*.

### Assembly of the three wheat homoeologues for each syntenic gene

We used two approaches to retrieve the wheat homoeologues of the Brachypodium syntenic genes. The first one made use of the flow sorted chromosome arm sequence database (IWGSC). We used BLAST homology searches (Altschul *et al.*, 1990) of the flow sorted chromosome arm sequence database using full length mRNA sequences of the Brachypodium syntenic genes as the query except for *TaELF3*, where the wheat mRNA GenBank accession number AK332315 was used as the query sequence. In some cases, sections of genes were missing from the chromosome arm sequence database and hence we used our second approach where we homology searched the unassembled reads of the Chinese Spring sequence database (Brenchley *et al.*, 2012) as for the chromosome arm survey sequence database. We then assembled the three wheat homologues for each of the 24 genes using Vector NTI sequence alignment tool as described for *T. aestivum FLOWERING LOCUS T 3* (*TaFT3*) in our earlier report (Zikhali *et al.*, 2014).

### Genome specific primer development

The primers were designed to have 100% match with 1DL sequences and to end with a 1DL specific base. The primers were also designed to contain as many mismatches with the 1AL and 1BL copies so as to exclude the 1AL and 1BL copies during PCR amplification. Markers were designed by re-sequencing 24 wheat genes on 1DL that are syntenous with *B. distachyon* chromosome 2 genes. The 1DL specific primers selectively amplified overlapping portions of 1DL gene copies while competitively excluding the A and B copies of the genes. At least one non-specific (amplified from all the three genomes) primer pair was designed for each gene and this was used as a control. We also designed 1DL specific primers for the region covering part of the BV211449 sequence (*Xbarc62*) and used these together with the primers developed by Song *et al.* (2005) for the same region to amplify PCR amplicons from *Aegilops tauschii* and the wheat varieties Chinese Spring, Savannah, Badger, Cadenza, Rialto, Avalon, and Charger.

### Amplification and sequencing of genes on 1DL

Amplicons were obtained from genomic DNA using standard PCR protocol and PCR reaction conditions, and detected by agarose electrophoresis as described in our earlier report (Zikhali *et al.*, 2014) for the 24 syntenous genes on 1DL. The amplicons were directly sequenced for Rialto, Avalon, Savannah, Charger, Spark, Badger, and Cadenza using ABI Big Dye Mix v3.1 (Applied Biosystems Inc.) under the manufacturer’s conditions, with products resolved on an ABI 3730 capillary electrophoresis instrument. For the genes *TaBradi2g14290* (*TaELF3*) and *TaBradi2g14340* (*TaMOT1*), we sequenced full length genomic sequences for the D copies of *TaELF3 and TaMOT1* as well as the *TaELF3-A1* and *TaELF3-B1* copies. For *TaBradi2g14330* (*TaFTSH4*), we sequenced most of the sequences except about 700 bases, which incuded exon 1 and part of intron 1 for both the B and D copies.

### Scoring single nucleotide polymorphisms in *TaFT3-D1*

Scoring of KASP single nucleotide polymorphisms (SNPs) to detect recombinants for the Spark X Rialto BC_2_F_5_ SSD populations was done as described by Zikhali *et al.* (2014) for *TaFT3-D1*. We also designed KASP assays (LGC) for: *TaBradi2g14790*, which we also used to identify recombinants in the Spark X Rialto SSD population; *TaELF3-D1*, which we mapped to Savannah X Rialto 1DL; and *TaELF3-B1*, which we mapped on the Avalon X Cadenza 1BL. We used the same KASP reagents as described for *Vrn-A1* (Diaz *et al*., 2012) and for *TaFT3-D1* (Zikhali *et al.*, 2014). Primer combinations for *TaBradi2g14790*, *TaELF3-B1*, *TaELF3-D1*, and *TaMOT1-D1* are shown in Supplementary Table S1 (available at *JXB* online).

### Genotyping Spark X Rialto NILs and SSD populations

The Spark X Rialto NILs and SSD populations were genotyped using the markers that we developed on 1DL as well as SSR markers *Xgdm111* and *Xcfd63*. We also scored the presence/absence of PCR amplicons on agarose gels for NILs, SSD, and DH population. Histograms were then used to classify lines as early or late. The markers also enabled us to identify recombinants between Spark and Rialto alleles on 1DL in the SSD populations.

### Comparative genomics exploiting synteny between Brachypodium and wheat

We used the wheat (IWGSC-based) pseudomolecule v3.3 (JIC) database and synteny between wheat and *B. distachyon* to determine the gene order on 1AL, 1BL, and 1DL. We used blast homology searches of the wheat (IWGSC-based) pseudomolecule v3.3 (JIC) using sequences linked to markers on 1DL and 1BL and retrieved the positions of these markers on each of the pseudomolecules. We then used the pseudo molecule positions to align the QTL on 1DL for Spark X Rialto, Avalon X Cadenza, and Savannah X Rialto, as well as the QTL on 1BL for Avalon X Cadenza with the 1AL region. We also aligned the *Eps*-A^m^1 (Bullrich *et al.*, 2002; Faricelli *et al.*, 2010) locus with the QTLs on the distal group 1 chromosomes.

### Gene expression

Expression studies and analysis of *TaGI* and *TaELF3* were carried out as described by Shaw *et al.* (2012) except that *norm2* (Forward primer AGCGATTTCCAGCTGCCTTC and reverse primer TGCGAAGAGGCCAGTCAGTC) was used as the expression reference instead of *RP15*. All above ground parts of 3-week-old plants grown under long days (16h light) at 16–18 °C in the light and 13–15 °C in the dark period were ground using a pestle and mortar. Two NIL pairs (NIL1 and NIL4, and NIL16 and NIL18) were selected for gene expression because these had the highest Rialto background relative to the other NILs (Zikhali *et al.* 2014). Samples were collected at 3 weeks at 3h intervals through a 24h period. Optimal genome specific qPCR assays could not be developed due to a relative paucity of homoeologous SNPs in the coding region, hence our limitation to total *TaELF3* expression using generic qPCR primers (Forward primer GTGGGATCGACAGACCTC and reverse primer CGACGCGTTCCTTCC).

### Genbank accession numbers for the genes sequenced in this study

1. *Triticum aestivum* Early Flowering 3-D1 (TaELF3-D1)

Rialto (KR055808), Avalon (KR055809), Charger (KR055810), Savannah (KR055811)

2. *T. aestivum* Early Flowering 3-B1 (TaELF3-B1)

Cadenza (KR082515), Spark (KR082517), Rialto (KR082518), Badger (KR082519), Savannah (KR082520), Avalon (KR082521), Charger (KR082522)

3. *T. aestivum* Early Flowering 3-A1 (TaELF3-A1)

Spark (KR082526), Savannah (KR082527), Rialto (KR082528), Avalon (KR082529), Cadenza (KR082530)

4. *T. aestivum* *MODIFIER OF TRANSCRIPTION 1*-D1 (TaMOT1-D1)

Rialto (KR082499), Avalon (KR082500), Charger (KR082501), Savannah (KR082502)

5. *T. aestivum* *MODIFIER OF TRANSCRIPTION 1*-B1 (TaMOT1-B1)

Charger (KR082507), Rialto (KR082508), Spark (KR082509), Avalon (KR082510),

Cadenza (KR082511), Badger (KR082512), Savannah (KR082513)

6. *T. aestivum* *FTSH PROTEASE 4*-D1 (TaFTSH4-D1)

Rialto (KR082535), Avalon (KR082536), Charger (KR082537), Savannah (KR082538)

7. *T. aestivum* *FTSH PROTEASE 4*-B1 (TaFTSH4-B1)

Avalon (KR082543), Charger (KR082544), Spark (KR082548), Badger (KR082549), Cadenza (KR082550), Rialto (KR082551), Savannah (KR082552)

## Results

### Satisfactory vernalization and photoperiod requirements in bread wheat

QTL profiles for DH populations show that the 1DL heading date QTL is an *Eps* effect because we detected QTLs after fully vernalizing the plants under LD and VLD for both DH populations but no significant QTL was detected under SD for both DH populations (Supplementary Fig. S1A,B available at *JXB* online). We detected QTLs for Spark X Rialto and Avalon X Cadenza (Supplementary Fig. S1A,B) at the same locus on the distal group 1 chromosome as was observed in the field (Griffiths *et al.*, 2009), and through QTL validation using NILs (Zikhali *et al.*, 2014). Since we did not detect a QTL for Malacca X Hereward on 5A, we concluded that 8 weeks of vernalization satisfied the vernalization requirement—given that a QTL with LOD score >22 was observed and shown to be due to copy number variation at the *Vrn-A1* when this DH population is vernalized for 4 weeks (Diaz *et al*., 2012).

### Position of *Xbarc62* on 1DL

**Fig. 1.**
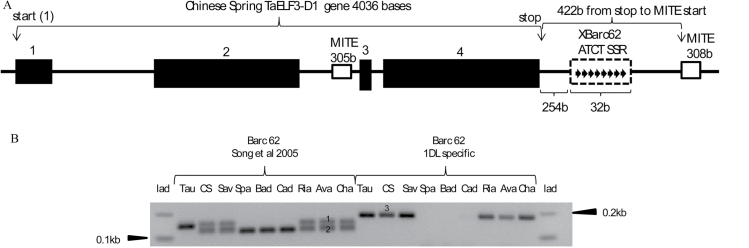
(A) Position of the *Xbarc62* SSR 254 bases downstream of the stop codon in the 3′UTR of the Chinese Spring *TaELF-D1* gene. The solid rectangles numbered 1–4 are the exons of the *TaELF3-D1* gene. The eight arrows in the dotted rectangle represent the eight ATCT repeats detected by the *Xbarc62* SSR marker (Song *et al.*, 2005). The two unshaded rectangles are miniature inverted transposable elements (MITEs) with similar sequences with sizes 305 and 308 bases, respectively. The start and stop represent the beginning (ATG) and end (TGA) respectively of the ORF. (B) Deletion of the *Xbarc62* locus in Spark (Spa), Badger (Bad), and Cadenza (Cad) as shown by the absence of the 129b (1) and 185b (3) size fragments when using the primers designed by Song *et al.* (2005) and the 1DL specific primers (this study), respectively. The 118b (2) fragment size is from the *TaELF3-A1* copy (see Supplementary Fig. S2 at *JXB* online).

The *Xbarc62* sequence is in the 3′-UTR of the *TaELF3-D1* gene and the ATCT SSR repeat detected by *Xbarc62* is 254b from the *TaELF3-D1* stop codon (Supplementary Fig. S2). The *Xbarc62* primers described by Song *et al.* (2005), are not 1DL specific as evidenced by the production of two PCR amplicons (129 and 118 bases) when these primers were used to amplify Chinese Spring (CS), Savannah (Sav), Rialto (Ria), Avalon (Ava), and Charger (Cha) but a single product (129b) from *A. tauschii* (Tau) (Fig. 1b). Sequencing revealed that the 118b size fragment (Fig. 1b), which is absent from *A. tauschii*, was from the A genome (1AL) copy (data not shown). When we used the 1DL specific primers, a single product (185b) was amplified from *A. tauschii*, Chinese Spring (CS), Savannah (Sav), Rialto (Ria), Avalon (Ava), and Charger (Cha) but there was no amplification from Spark, Badger, and Cadenza (Fig. 1b).

The *Xbarc62* SSR marker does not amplify a D copy amplicon in Spark, Badger, and Cadenza and is scored as a null (data not shown). This suggested a deletion or other disturbance of the sequences in these varieties. The *Xbarc62* primers by Song *et al.* (2005) amplify from both the A and D copies because they do not distinguish the two homoeologues (Supplementary Fig. S2 at *JXB* online). We showed this by aligning the GenBank accession BV211449 sequence, which contains the ATCT *Xbarc62* repeat, with sequences from *A. tauschii ELF3* (*AtELF3*), *T. urartu ELF3* (*TuELF3*), and the three wheat homoeologues of the *TaELF3* gene (Supplementary Fig. S2).

**Fig. 2.**
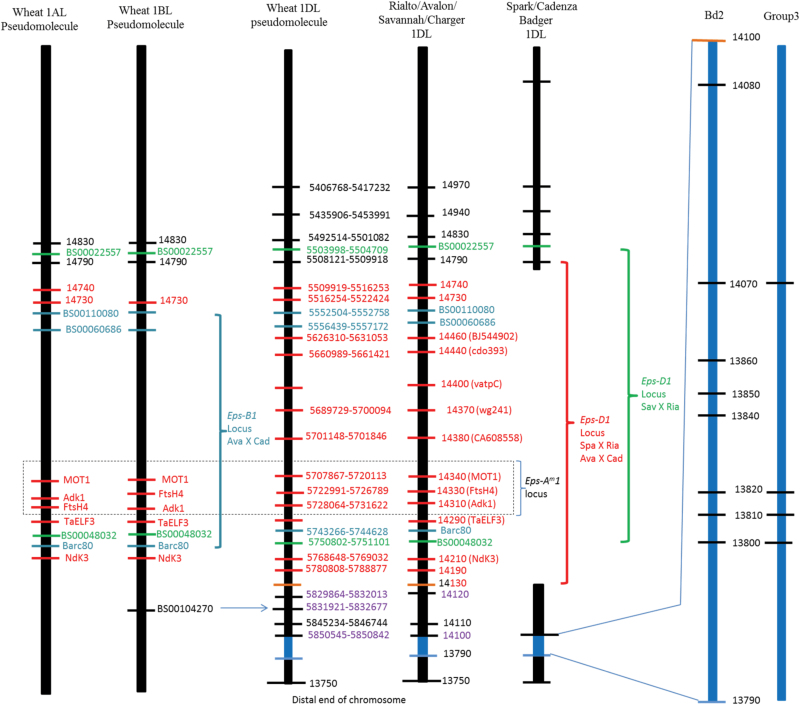
Schematic presentation of the *Eps-B1* locus for Avalon X Cadenza (QTL confidence interval between SSR marker *XBarc80* and KASPar markers XBS0011080 and XBS00060686, *Eps-D1* (deletion of several genes on 1DL of Spark), *Eps-D1* for Savannah X Rialto (no deletion) QTL confidence interval between KASPar markers XBS00048032 and XBS00022557. The dotted rectangle marks the Eps-Am1 locus and the two proposed candidates *MOT1* and *FTSH4* (Faricelli *et al.*, 2010). The blue and green horizontal numbers and letters indicate SSR marker (*XBarc80*) and KASPar markers beginning with BS. The black vertical bars indicate the 1AL, 1BL, and 1DL wheat pseudomolecules from the wheat (IWGSC-based) pseudomolecule v3.3 (JIC) database and the region is collinear with Brachypodium chromosome 2. The red or black numbers on the right of Rialto 1DL next to the red or black horizontal bars, respectively, indicate the Brachypodium chromosome 2 gene numbers syntenic with wheat 1DL. The red numbers and letters on the right of Rialto/Avalon/Savannah/Charger 1DL indicate the genes deleted from Spark while the black numbers show those outside the 1DL deletion. The purple coloured numbers on the right side of wheat 1DL psuedomolecule and Rialto/Avalon/Savannah/Charger 1DL denote the genes between *TaBradi2g14130* and *TaBradi2g13790* that have not been checked if they are in the deletion but are likely to be outside the 1DL deletion. The gap on the vertical bar labelled Spark/Cadenza/Badger 1DL indicates the 1DL deletion. The blue vertical bars (Bd2 and group 3) are used to illustrate that all the eight brachypodium genes (13800, 13810, 13820, 13840, 13850, 13860, 14070, and 14080) between the predicted genes *Bradi2g13790* (designated 13790 on Fig. 2) and 14130 do not match the wheat group 1 genes but four of these genes match the group 3 chromosome genes. The gene *TaBradi2g14130* (14130) is labelled in black and red because part of this gene was amplified and sequenced from Spark while the rest of the gene seems to be deleted.

Having shown that the *Xbarc62* SSR marker was linked to the *TaELF3-D1* gene (Supplementary Fig. S2), we designed primers specific for coding sequence from *TaELF3*-*D1.* Spark, Badger, and Cadenza did not yield PCR amplicons while Rialto, Avalon, Savannah, and Charger did. The same pattern was also observed for PCR primers based on the Brachypodium syntenic genes flanking *TaELF3*, suggesting that there was a deletion that includes several genes for Spark and Cadenza while Rialto, Avalon, Savannah, and Charger are intact in this region (Fig. 2).

### Defining the *Eps-D1* (1DL deletion) and *Eps-B1* loci

**Fig. 3.**
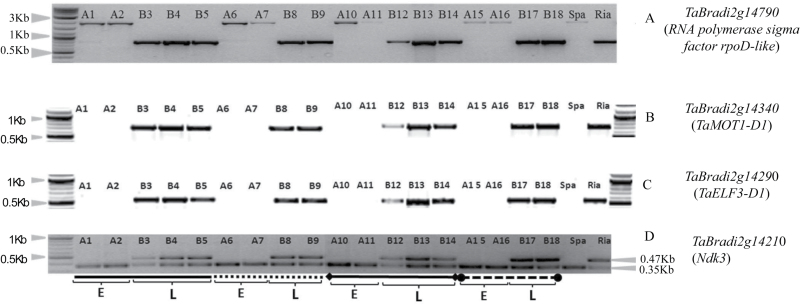
The genotypes of the Spark X Rialto 1DL NILs using markers *TaBradi2g14790* (A), *TaBradi2g14340* (B), *TaBradi2g14290* (C), and *TaBradi2g14210* (D). The letters A and B, which are followed by numbers, indicate the NILs carrying the Spark and Rialto allele, respectively. Spa and Ria are Spark and Rialto wheat cultivars, respectively. The solid horizontal line from A1 to B5 designates the first NIL pair, the dotted horizontal line (A6–B9) designates the second NIL pair, the solid horizontal line flanked by diamond shapes (A10–B14) is the third NIL pair and the dashed horizontal line flanked by circles (A15–B18) is the fourth NIL pair. The letters E and L at the bottom of the Figure indicate early flowering (E) and late flowering (L), respectively, of the NILs. The fragment size markers are shown in kilobases (kb). We showed the flowering time differences both in the field and controlled environments for these NILs in our earlier report (Zikhali *et al.*, 2014).

Based on the synteny with Brachypodium and the wheat 1DL pseudomolecule, there is no gene on wheat 1DL between *TaBradi2g14790* and *TaBradi2g14740* (Fig. 2; Supplementary Table S2 at *JXB* online). The gene *TaBradi2g14790* is outside the 1DL deletion on the proximal side (Figs 2 and 3). The marker segregation shown on Fig. 3A is due to an insertion in the *TaBradi2g14790* gene for Spark. We developed a KASP assay for *TaBradi2g14790* that we used to identify recombinants. Since all the 10 PCR primers that span *TaBradi2g14740* do not amplify from Spark (Supplementary Fig. S3.2H at *JXB* online), this suggests the proximal deletion breakpoint is in the inter-genomic region between *TaBradi2g14790* and *TaBradi2g14740*. The distal deletion breakpoint may be in the gene *TaBradi2g14130*, given that we sequenced part of this gene but the rest seems deleted from Spark.

The 1DL subtelomeric deletion spans the equivalent of the entire *Eps-A* ^*m*^ *1* locus (Faricelli *et al.*, 2010; Fig. 2), which makes *TaMOT1-D1* and *TaFTSH4-D1* potential candidates for *Eps-D1*. The deletion also contains a homologue of the *Arabidopsis* circadian clock gene *ELF3* designated *TaELF3-D1* (Higgins *et al.*, 2010). It has been suggested but not proven that *Eps-A* ^*m*^ *1* was orthologous with QTLs on *T. aestivum* group 1 chromosomes (Griffiths *et al.*, 2009), and our results show that the QTLs on 1DL (Spark X Rialto, Avalon X Cadenza, Savannah X Rialto) and 1BL (Avalon X Cadenza) have QTL confidence intervals that span the region equivalent to the entire *Eps-A* ^*m*^ *1* locus (Fig. 2; Supplementary Fig. S4A, B).

The *TaELF3*-*D1* gene was not assigned a position on the wheat 1DL pseudomolecule (Fig. 2) but we placed it between *XBarc80* and *Adk1* on Rialto 1DL because we mapped this gene between markers *XBS00048032* and *XBS00022557* (markers shown in green in Fig. 2) in Savannah X Rilato 1DL (Supplementary Fig. S4B at *JXB* online). We also mapped *TaELF3-B1* between *XBarc80* and *XBS00110080/XBS00060686* in Avalon X Cadenza (Supplementary Fig. S4B). Although both *XBS00110080/XBS00060686* were not assigned on 1BL pseudomolecule (Fig. 2), these markers were placed distal to *TaBradi2g14730* because both are distal to *TaBradi2g14730* on 1AL and 1DL pseudomolecules. The *TaELF3*-*A1* and *TaELF3-B1* genes are between markers *XBS00048032* and *Adk1* on both 1AL and 1BL pseudomolecules (Fig. 2). Since *Adk1*, *XBarc80*, and *XBS00048032* were all assigned positions on the 1AL, 1BL, and 1DL pseudomolecules, the *TaELF3-D 1* position on 1DL was determined to be between *XBarc80* and *Adk1* (Fig. 2).

### *Eps-D1* NIL genotypes

We showed previously that the 1DL NILs carrying the Spark alleles at this locus were all early flowering relative to those with the Rialto allele, both in the field and controlled environments (Zikhali *et al.*, 2014). Our genotyping results for the same 1DL NILs (Fig. 3) suggest that the deletion is the likely cause of the flowering time differences between the NIL pairs. The expected amplicon size for *TaBradi2g14210* was the 470bp amplicon, which is absent from Spark, and the 1DL NILs with the Spark allele (Fig. 3D). The 0.35kb amplicon was sequenced for Spark and did not match the group 1 chromosomes while the 470bp amplicon was sequenced and matched the 1DL gene copy. This suggested that the *TaBradi2g14210* gene is also deleted in Spark.

### Phenotyping and genotyping the *Eps-D1* BC_2_F_5_ populations

**Fig. 4.**
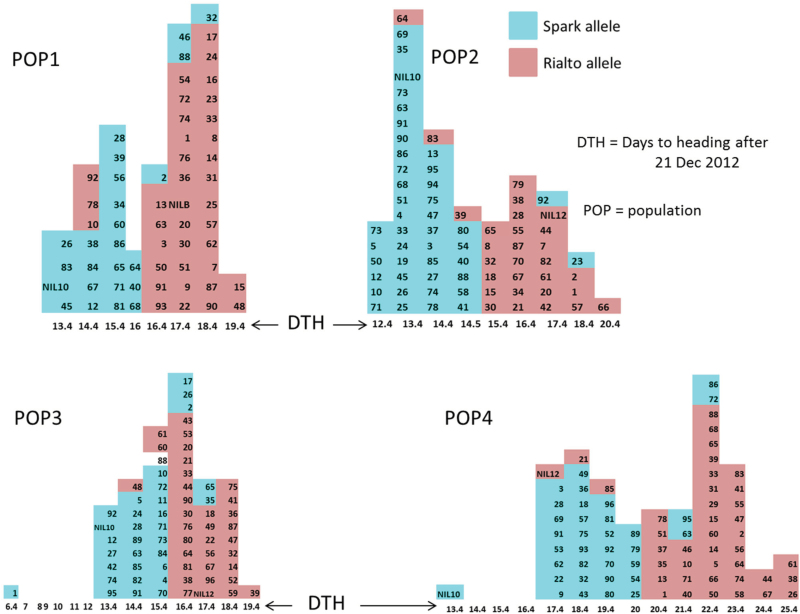
Days to ear emergence measured at growth stage 55 (Zadoks *et al.*, 1974) of four independent Spark X Rialto Backcross 2 (BC2) SSD populations (POP1–POP4) scored at F5 (BC2F5). The two NILs, NIL10 and NIL12, carrying the Spark and Rialto 1DL alleles, respectively, were used as controls and grown together with SSD populations. Key: DTH was measured at growth stage 55 (Zadoks *et al.*, 1974) shown by the arrows pointing at the uncoloured numbers. The coloured numbers are the individual SSD lines. POP1, POP2, POP3, and POP4 are the four Spark X rialto 1DL SSD populations at BC_2_F_5_. Spark and Rialto alleles are the 1DL deletion and intact chromosomes, respectively.

We observed a bimodal distribution of lines carrying the deletion and those with an intact portion of the chromosome for SSD populations 1, 2, and 4 (POP1, POP2, and POP4) (Fig. 4). The division of population 3 (POP3) into early and late groups becomes apparent when classified according to deletion genotype (Fig. 4). Most lines carrying the deletion have an early flowering phenotype and this was consistent with the 1DL NILs, which we used to validate this QTL in an earlier report (Zikhali *et al.*, 2014).

The following lines POP1 (10, 78, 92 and 2, 32, 46 and 88), POP2 (39, 64 and 83 23, 65 and 92), POP3 (13 (48, 60, 61, 2, 17, 26, 35, 65), and POP4 (21, 85, 63, 72, 86 and 95) run against the trend and are hereafter referred to as outliers. The genotypes of the outliers are shown on Supplementary Fig. S5. Line P1_78 has a Rialto 1DL arm, while lines P2_23 and P3_65 have Spark 1DL arms with no recombination, hence these three outliers are not due to recombination on 1DL (Supplementary Fig. S5). The flowering time shift observed for POP4, which flowers 5 d later than the other populations, as well as the very early flowering observed for line 1 from POP3 (Fig. 4), suggest that there could be a genetic background effect—given that our SSD populations were self-fertilized up to BC2F5 with expected Spark background of 12.25%. It is interesting to note that the gene responsible for the observed delay in heading date in POP4 relative to POP1, POP2, and POP3 is independent of the 1DL *Eps* effect (Fig. 4) and just delays the flowering of the whole population by 5 d relative to the other three populations (Fig. 4).

### Recombinants suggest the 1DL deletion may contain the candidate for *Eps-D1*

**Fig. 5.**
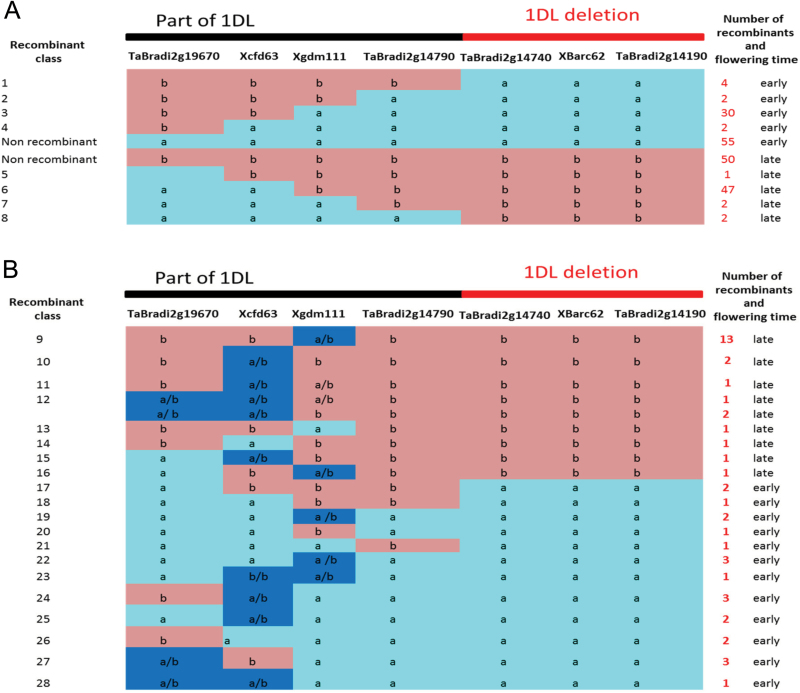
(A) Single recombinants at the 1DL QTL interval that segregate for late flowering and early flowering in the BC_2_F_5_ SSD lines of a cross between Spark and Rialto. Recombinant classes 1–4 are early flowering while recombinant classes 5–8 are late flowering. The data show that the earliness allele is distal to the *TaBradi2g14790* marker. (B) Recombinants containing heterozygous regions as well as double recombinants at the 1DL QTL interval that segregate for late flowering and early flowering in the BC_2_F5 SSD lines of a cross between Spark and Rialto. Recombinant classes 9–14 are late flowering while recombinant classes 15–28 are early flowering. Key a=Spark 1DL *Eps-D1* allele, b=Rialto 1DL allele and a/b represents heterozygous for a and b. The data also shows that the earliness allele is linked with the 1DL deletion.

One hundred and thirty-five lines that are recombinants on 1DL (Fig. 5A,B) suggest that the *Eps-D1* effect is distal to the marker *TaBradi2g14790*, which Brachypodium gene content would predict to be the last intact gene in the proximal side of the deletion (Fig. 2). The distal end of the deletion is near the telomeric region and we were unable to identify recombinants on the distal end of the deletion.

### Prioritization of candidates for *Eps-D1* by exploiting homoeologous relationships

It is not possible to genetically dissect the putative *Eps-D1* deletion and so all of the genes within it have equal standing as candidate genes. We decided to use homoeologous relationships and other populations with QTL in equivalent locations to try to prioritize the list of candidates. A QTL on 1BL identified in Avalon X Cadenza, which we named *Eps-B1*, is likely to be homoeologous with *Eps-D1* (Fig. 2). In addition, *Eps-D1* is orthologous with *Eps*-*A* ^*m*^ *1* (Faricelli *et al.*, 2010) since the equivalent of the entire *Eps*-*A* ^*m*^ *1* confidence interval is spanned by the *Eps-D1* deletion (Fig. 2). We also detected a QTL for Savannah X Rialto DH population in the region that spans the *Eps-D1* deletion and *Eps*-*A* ^*m*^ *1* equivalent region; even though neither of these varieties carries the deletion (Fig. 2). We used these homoeologous relationships to try to prioritize/rank candidates. Out of the 33 syntenic genes in the 1DL deletion (Supplementary Table S2) we prioritized *TaMOT1-D1*, *Ta*FTSH4*-D1*—based on previous studies in *T. monococcum* (Faricelli *et al.*, 2010), and *TaELF3-D1* genes as possible candidates for *Eps-D1* and *Eps-B1*. The *TaELF3-D1* gene was prioritized because its homologues have been reported to affect flowering time in *Arabidopsis* (Dixon *et al.*, 2011), *Oryza sativa* (Matsubara *et al.*, 2012), *Hodeum valgare* (Faure *et al.*, 2012; Zakhabekova *et al*., 2012), *Zea mays* (Bate and Aukerman, 2011), and the legumes lentil and pea (Weller *et al.*, 2012). Sequencing the homoeologous copies of these three genes in the wheat cultivars Avalon, Rialto, Charger, Savannah, Spark, Cadenza, and Badger revealed the mutations outlined below. For *TaELF3-D1*, two SNPs which are silent mutations in exon 2 and exon 4 (at positions 1746 and 3343 from the start codon) for Savannah (early allele) distinguishes it from the wild type (Table 1; Supplementary Fig. S6A). We designed a KASP assay for the *TaELF3-D1* exon 4 SNP (Table 1; Supplementary Fig. S6A), which we used to map the gene in the Savannah X Rialto DH population (Supplementary Fig. S4B). For *TaELF3-B1*, where Avalon carries an early allele, a SNP in exon 4 (position 3432 from the start codon for Avalon) changes a conserved glycine to serine (Supplementary Fig. S7) and distinguishes Avalon from the wild type (Table 1; Supplementary Fig. S6B). We designed a KASP assay for the *TaELF3-B1* and used it to map the gene in the Avalon X Cadenza DH population (Supplementary Fig. S4A).

**Table 1.** *Mutations at* TaELF3-D1, TaELF3-B1, TaMOT1-D1, TaMOT1-B1, TaFTSH4-D1, *and* TaFTSH4-B1 *and their association with heading date phenotype at the* Eps-D1 *and* Eps-B1 *loci* No mutation in the ORF means there was no SNP deletion or insertion from the start codon (ATG to the stop codon TGA or TAG). Spark/Cadenza or Rialto/Avalon means both varieties Spark and Cadenza or Rialto and Avalon have the same mutations for the three genes *TaELF3-D1*, *TaMOT1-D1,* and *TaFTSH4-D1* except for *TaMOT1-D1* where Rialto only has a SNP in exon 6.

**Variety**	**Gene**	**Polymorphism**	**Allele effect**
Spark/	*TaELF3-D1*	Whole gene deleted	Early heading
Cadenza	*TaMOT1-D1*	Whole gene deleted	
	*TaFTSH4-D1*	Whole gene deleted	
Savannah	*TaELF3-D1*	Silent SNPs in exons 2 & 4	Early heading

	*TaMOT1-D1*	No mutation in the ORF	
	*TaFTSH4-D1*	No mutation in the ORF	
Rialto/	*TaELF3-D1*	No mutation in the ORF	Late heading
Avalon	*TaMOT1-D1*	24 ATT repeats (intron1) SNP intron 6 (Rialto)	
	*TaFTSH4-D1*		
		Exon 7 SNP changes serine to leucine	
Avalon	*TaELF3-B1*	Exon 4 SNP changes glycine to serine	Early heading

	*TaMOT1-B1*	No mutation in the ORF	
	*TaFTSH4-B1*	No mutation in the ORF	
Cadenza	*TaELF3-B1*	No mutation in the ORF	Late heading
	*TaMOT1-B1*	No mutation in the ORF	
	*TaFTSH4-B1*	Premature stop codon due to 17b deletion	
		in exon 3	

For *TaELF3-A1*, seven mutations were observed along the gene (Supplementary Fig. S6C). The first is a GGATT SSR repeat with Spark having 9 of these GGATT repeats, Savannah has 8, Chinese Spring has 7, and the wild type including *T. urartu* has 6 repeats. Spark has a C/T SNP that distinguishes it from the wild type at position 614 (Supplementary Fig. S6C). Positions 1359, 1407, 1507, and 2798 have SNPs that result in silent mutations and distinguish Spark/Savannah from wild type, Savannah from wild type, Spark/Savannah from wild type, and Spark from wild type, respectively (Supplementary Fig. S6C). The SNP at position 2703 in exon 4 of *TaELF3-A1* changes the wild type glycine to tryptophan for Spark/Savannah (Supplementary Fig. S6C). In spite of the SNPs on *TaELF3-A1*, we did not detect a QTL on 1AL in the populations we used. Chinese Spring and all the wheat varieties we used in this study have an approximately 1.25 and 0.95kb deletion in the intergenic region between *TaELF3-A1* and the upstream gene TaBradi2g14280 relative to the B and D copies respectively.

For *TaMOT1-D1*, two mutations were observed; the first starting at position 93 (intron 1) from the start codon where Avalon/Rialto have 24 ATT repeats and the wild type has 22, and a SNP at position 2702 (intron 6) which distinguishes Rialto from the wild type (Supplementary Fig. S6D). We were unable to sequence the whole of exon 1 and part of intron 1 but sequenced the rest of the gene for both *Ta*FTSH4*-D1* and *Ta*FTSH4*-B1.* For *Ta*FTSH4*-D1*, a SNP at position 3955 changes a conserved serine to leucine for Rialto/Avalon (Table 1; Supplementary Fig. S6E). For *TaFTSH4-B1*, a 17 base deletion in exon 3 introduces a premature stop codon for Spark, Badger, Cadenza, Rialto, and Savannah, and this distinguishes them from Charger and Avalon, which have an intact exon 3 (Table 1; Supplementary Fig. S6F). There were no mutations detected in the coding sequence in the *TaMOT1-B1* gene for all the varieties we sequenced.

### *TaGIGANTEA (TaGI*) and *TaELF3* expression analysis

**Fig. 6.**
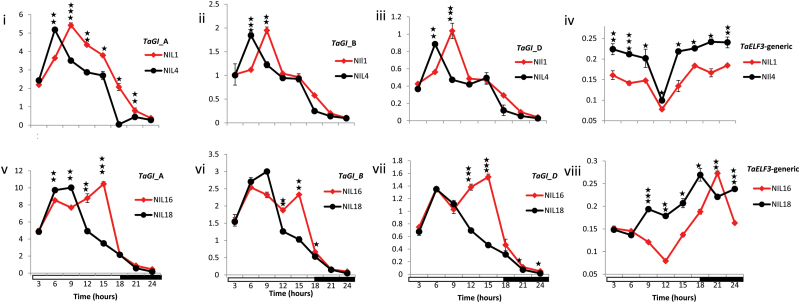
Expression patterns of the evening—loop gene *TaGI* A, B, and D homoeologues and that of the T*aELF3* genes (generic primer amplifying all three A, B and D homoeologues) in the *Eps-D1* mutants (NIL1 and NIL16) (red diamonds and lines) and the wild type (NIL4 and NIL18) (black circles and lines). The vertical axis is the relative expression of the genes against the house keeping gene *norm2*. Plants were grown under long days (16h light and 8h darkness) for 21 d and sampled at 3h intervals beginning at 0300 hrs which was 3h after turning on the lights, except samples taken at 2100 hrs (21), which were sampled 2h 45min after the seventh sample (18) to ensure that these samples were taken during darkness. Lights were turned off at 1600 hrs such that three samples were taken during the dark period indicated by the black solid bar on the horizontal axis. The stars indicate significant difference in expression ***P<0.001, **P<0.01 and *P<0.05 using the Student’s *t*-test for each time point. The error bars are the standard error of the mean of two technical replicates derived from a pool of three plants.

Because we considered the deletion of *TaELF3-D1* within *Eps-D1* locus to be a very likely cause of early flowering in Spark, we undertook expression analysis of *TaELF3* to show that loss of the D copy did indeed result in reduced total expression, discounting compensation from other genomes or non-expression of the intact copy. We also looked at expression of *TaGI* as *TaELF3-D1* homologues are known to be direct repressors of *GI* in a number of species. Figure 6 shows that in both NIL pairs *GI* expression was increased with the presence Spark *Eps-D1* allele relative to Rialto later in the light period. This pattern was observed for all three homoeologues in two independent NIL genetic backgrounds (Fig. 6). For *TaELF3*, total expression was consistently higher with the presence of the *Eps-D1* Rialto allele in NIL4. For NIL pair 16/18 Spark alleles were associated with reduced *TaELF3* expression except for time-points 5 and 7.

## Discussion

This study shows that bread wheat *Eps* genes, still segregating in the elite gene pool, can be isolated as Mendelian factors and fine mapped. This has enabled us to define *Eps-D1* and show that a deletion on 1DL is responsible for the early flowering phenotype of Spark. This means that *Eps* genes can be defined and positionally cloned in the same way as the first generation of flowering time genes *Ppd* (Turner *et al.*, 2005) and *Vrn* (Yan *et al.*, 2003) genes were. This study used elite winter wheat varieties Spark, Rialto, Avalon Cadenza, and Savannah, and we show, using NILs and SSD populations that it is possible to fine map these effects directly in hexaploid wheat.

Lin *et al.* (2008) and Griffiths *et al.* (2009) both speculated that the QTLs on the distal end of group1 chromosomes were homoeologues of the *T. monococcum Eps*-*A* ^*m*^ *1* (Bullrich *et al.*, 2002; Valarik *et al.*, 2006; Faricelli *et al.*, 2010). Results from this study show that the *Eps*-*A* ^*m*^ *1* is at the same locus as the *T. aestivum Eps-D1* and *Eps-B1* locus given that the QTL confidence interval for *Eps*-*A* ^*m*^ *1* is in the 1DL deletion that this study showed to be tightly linked with the heading date *Eps* QTL (Figs 2–5).

### Probable candidates for the *Eps* gene in the 1DL deletion

The results from this study suggest that the *Eps-D1* effect is likely due to a deletion that includes several genes. All of the genes in the deletion are potential candidates for the *Eps* effect and the possibility of more than one gene being responsible is not ruled out. Deletion mutations of large portions of chromosomes (Shitsukawa *et al.*, 2007; Distelfeld and Dubcovsky, 2010), single genes (Faure *et al.*, 2012), or portions of genes (Yan *et al.*, 2003; Fu *et al.*, 2005; Wilhelm *et al.*, 2009) have been shown to cause variation in flowering time.

### Possibility that MODIFIER OF TRANSCRIPTION 1 (*MOT1*) is the candidate

However, among the deleted genes there are some candidates which stand out. These are *MODIFIER OF TRANSCRIPTION 1* (*MOT1*) and *FTSH PROTEASE 4* (*FTSH4*)—the suggested candidates for the *Eps*-*A* ^*m*^ *1* (Faricelli *et al.*, 2010).

The parental lines Spark and Cadenza carry the 1DL deletion (Fig. 2) which makes *MOT1-D1* a potential candidate for *Eps-D1* given that the same QTL is observed at the same locus for the Spark X Rialto and Avalon X Cadenza DH populations (Supplementary Fig. S1). However, neither Savannah nor Rialto carries the 1DL deletion but a flowering QTL was observed at the same locus as that of Spark X Rialto and Avalon X Cadenza DH populations (Fig. 2; Supplementary Fig. S4B). Sequencing the *TaMOT1-D1* gene revealed no mutations in all the 28 exons but two (introns 1 and 6) mutations differentiate Rialto from Savannah (Table 1). Given that both Rialto and Avalon have the ATT repeat mutation in *TaMOT1-D1*, intron 1 can only be the causal mutation if it is a gain of function mutation because this gene is deleted for both Spark and Cadenza which segregate with Rialto and Avalon, respectively, for the 1DL effect. The SNP in intron 6 is unlikely to be the cause given that it is only present in Rialto but absent from Avalon.

### FTSH PROTEASE 4 (*FTSH4*) as possible candidate

The other gene that was suggested as a candidate for *Eps*-*A* ^*m*^ *1* is *FTSH4.* The *Arabidopsis FTSH4* mutants flower a week later than wild type (Gibala *et al.*, 2009). However, in *T. monococcum*, there was no amino acid substitution in the predicted *FTSH4* protein sequences of the lines segregating for the *Eps*-*A* ^*m*^ *1* (Faricelli *et al.*, 2010). Again there were no significant *FTSH4* transcript differences between NILs segregating for *Eps*-*A* ^*m*^ *1* (Faricelli *et al.*, 2010). In this study, sequencing *TaFTSH4-D1* revealed that Rialto/Avalon and Savannah segregate for a mutation that changes a conserved serine to leucine in Rialto and Avalon (Table 1; Supplementary Fig. S6A).

From Table 1, it seems *FTSH4* is a flowering promoter—given that loss of the gene *TaFTSH4-B1* in Cadenza is linked with late phenotype while the wild type *FTSH4-B1* in Avalon is linked with the early phenotype. In, Rialto, *TaFTSH4-D1* mutation is linked with the late phenotype while Savannah has no mutation and is linked with an early phenotype (Table 1). These results from the two homoeologues of *FTSH4* are consistent with *FTSH4* being a flowering promoter—as in *Arabidopsis* (Gibala *et al.*, 2009)—and make *FTSH4-B1* and *FTSH4-D1* likely candidates for *Eps-B1* and *Eps-D1*. However, when we also consider that *TaFTSH4-D1* and *TaELF3-D1* are both deleted from Spark and Cadenza, and the deletion is linked with the early phenotype (Figs 2–5; Table 1), the candidature of *TaFTSH4-D1* is questionable as its loss should result in late flowering.

For the *Eps-B1* locus, sequencing of the *TaFTSH4-B1* gene revealed that Cadenza has a deletion in exon 3, which introduces a premature stop codon, while Avalon has got an intact exon 3 (Table 1. However, even though Charger and Badger segregate for the same mutation as Avalon and Cadenza on 1BL, no *Eps-B1* QTL was detected both in the field (Griffiths *et al.*, 2009) and controlled environments for the Charger X Cadenza DH population. This brings to question the possibility that *TaFTSH4-B1* is a candidate for *Eps-B1* although it is possible that a background effect could be masking the effect in the Charger X Badger population.

It should also be pointed out here that the study in *Arabidopsis* showed that *FTSH4* loss did not affect growth under adequate photoperiod but only affected late rosette development under limiting photoperiod when the plants were grown in short days (Gibala *et al.*, 2009). This was different from the effect caused by *Eps-D1* and *Eps-B1*, where the effect was stronger under long day conditions but not detectable under short days for both the Spark X Rialto and Avalon X Cadenza DH populations (Supplementary Fig. S1).

### Possibility that *TaELF3-D1* is the candidate for *Eps-D1*

In this study, *TaELF3-D1*, a circadian clock gene, is suggested as a possible candidate for *Eps-D1* in addition to *MOT1-D1* and *FTSH4-D1* because it is contained within the subtelomeric deletion and has been shown to affect flowering in a number of species. The *ELF3* gene is a repressor of flowering time in at least six species and one would predict that loss of function mutations like premature stop codons or deletion of the entire gene would result in an early flowering phenotype for wheat. Our results using NILs (Fig. 3; Zikhali *et al.*, 2014) and SSD populations (Figs 4 and 5) for crosses between Spark and Rialto show that the deletion is linked with an early flowering phenotype and this is consistent with loss of *ELF3* hence *TaELF3-D1* is a likely candidate for *Eps-D1*. Here we offer support at the expression level for *TaELF3-D1* as a candidate, because *TaELF3* total expression is reduced when the early Spark allele is present and this is accompanied by increased expression of *TaGI* (Fig. 6). The Spark allele has the 1DL deletion, which includes *TaELF3-D1*, and since there is no *TaELF3-D1* contributed by the Spark allele, we attribute the significant difference in the total *TaELF3* expression to this deletion. Again our results suggest that there is no compensation from the other two genomes since the difference in total *TaELF3* expression is significantly different in two independent NIL pairs (Fig. 6). All the three homoeologues of *TaGI* have higher expression later during the day in the *Eps-D1* mutants relative to the wild type as would be expected for an *ELF3* mutant since *ELF3* is a repressor of *GI* (Higgins *et al.*, 2010; Dixon *et al.*, 2011; Faure *et al.*, 2012; Zakhrabekova *et al.*, 2012). Further studies will be needed to determine why the loss of only one genome leads to early flowering, but at this stage we can only speculate that this may be a dosage effect.

A patent in maize (Bate and Aukerman, 2011) showed that manipulating *ELF3* can help to increase maize yield. The patent shows that over-expressing the *Zea mays ELF3* (*ZmELF3*) gene enabled the plants to be grown at high density. This was because over-expressing *ZmELF3* suppressed the shade avoidance response by enabling plants to tolerate limited light. The functioning of the *ZmELF3* in delaying flowering is consistent with observations in our study where loss of *TaELF3-D1* by deletion of the gene (Spark and Cadenza) results in early flowering relative to the plants with an intact gene which are later flowering. Furthermore, Boden *et al.* (2014) showed that the barley *EARLY FLOWERING 3* gene suppresses gibberellin biosynthesis and that plants with constitutive gibberellin biosynthesis flowered earlier than mutants. Again this result is consistent with results from this study which suggest that *Eps-D1* is due to a floral repressor, given that the 1DL deletion is associated with early flowering (Figs 2–5).

It has not escaped our attention that *Eps-D1* is due to a deletion that includes several genes, so it is quite possible that another flowering repressor gene or a micro RNA could be among the deleted genes and act in addition to the candidates we characterized in this study. For Savannah X Rialto *Eps-D1* locus (Supplementary Fig. S4B), no candidate mutations in the gene were observed but there were two silent mutations in exons 3 and 4 in Savannah (Supplementary Fig. S6A). Epigenetic control for *TaELF3-D1* or other mutations in the promoter that we did not sequence may account for the Savannah X Rialto *Eps-D1* effect. Further studies can be done in future to test this hypothesis.

### Possibility that *TaELF3-B1* is the candidate for *Eps-B1*

Sequencing *TaELF3-B1* also showed that this gene is a plausible candidate for *Eps-B1* because Avalon has a mutation that changes a conserved glycine to serine in exon 4 (Supplementary Fig. S6A). The mutant *TaELF3-B1* (Avalon) would be predicted to result in early flowering relative to the wild type Cadenza and our results suggest that this is the case (Table 1).

However, Charger and Badger are polymorphic for the 1DL deletion (Figs 1B and 2) but there was no *Eps-D1* QTL detected for this population in the field (Griffiths *et al.*, 2009) and in the controlled environments in this study, as was observed for *Eps-B1*. We had expected this population to behave like the Spark X Rialto and Avalon X Cadenza DH populations, which segregate for *Eps-D1* (Supplementary Fig. S1). Given that *TaMOT1-D1*, *TaFTSH4-D1*, and *TaELF3-D1* are all deleted from Badger, we tested the hypothesis that the candidate gene among the three would have a loss of function mutation for Charger that would account for the unavailability of the *Eps-D1* effect in the Charger X Badger population by sequencing the three genes for Charger. However, we did not detect mutations in Charger for all the three genes in their coding sequences. It is possible that there could be promoter mutations that we did not sequence in the three genes that may account for why Charger X Badger does not segregate for *Eps-D1*. It is also possible that Charger X Badger population behaves like the outliers for *Eps-D1* (Supplementary Fig. S5). Further studies in future may resolve this anomaly.

Apart from the anomaly observed for Charger, which affects the candidature of all the three genes we prioritized, *TaELF3-D1*, *TaMOT1-D1*, *and TaFTSH4-D1*, we ranked *TaELF3-D1* higher than *TaFTSH4-D1* and *TaMOT1-D1* as a possible candidate for *Eps-D1* because higher expression of *TaGI* later during the day in the NILs carrying the *Eps-D1* deletion and reduced total *TaELF3* expression coupled with early flowering relative to the wild type is consistent with an *ELF3* mutant. In addition to that, the mutations at *TaELF3* explain the phenotypes at both *Eps-D1* and *Eps-B1*, while mutations at both *TaMOT1* and *TaFTSH4* at best explain the phenotype at one locus.

## Supplementary data

Supplementary data are available at *JXB* online.

Fig. S1. *Eps-D1* QTLs.

Fig. S2. Position of the *Xbarc62* SSR marker on 1DL.

Fig. S3. Agarose gel pictures showing the genes used to define the 1DL deletion.

Fig. S4. Chromosomal location of *Eps-B1* QTL and Savannah X Rialto *Eps-D1* QTL.

Fig. S5. The genotypes of the outliers in the Eps-D1 region

Fig. S6. Schematic diagrams showing the positions of SNPs for the genes *TaELF3-D1*, *TaELF3-B1*, *TaELF3-A1*, *TaMOT1-D1*, *TaFTSH4-D1*, and *TaFTSH4-B1*.

Fig. S7. Alignment of *ELF3* proteins from different species showing conserved amino acid change in *TaELF3-B1*.

Table S1. The 40 syntenous *B. distachyon* genes used to define the 1DL deletion.

Table S2. KASP primer combinations for *TaBradi2g14790*, *TaELF3-B1*, *TaELF3-D1* and *TaMOT1-D1*

Supplementary Data
