# Supplementary material for: Delimitation of the Earliness per se D1 (Eps-D1) flowering gene to a subtelomeric chromosomal deletion in bread wheat (Triticum aestivum)
Source: J Exp Bot. 2015 Oct 17;67(1):287–99. doi: 10.1093/jxb/erv458 (PMC4682435; doi:10.1093/jxb/erv458)
Supplement: Supplementary Data [file supp_67_1_287__index.html]

Delimitation of the Earliness per se D1 (Eps-D1) flowering gene to a subtelomeric chromosomal deletion in bread wheat (Triticum aestivum) — Supplementary Data 

# Delimitation of the *Earliness per se D1* (*Eps-D1*) flowering gene to a subtelomeric chromosomal deletion in bread wheat (*Triticum aestivum*)

## Supplementary Data

Data files

- Supplementary\_Tables\_S1\_S2\_Figures\_S1\_S7.pdf - Supplementary Data
