# Supplementary material for: Delimitation of the Earliness per se D1 (Eps-D1) flowering gene to a subtelomeric chromosomal deletion in bread wheat (Triticum aestivum)
Source: J Exp Bot. 2015 Oct 17;67(1):287–99. doi: 10.1093/jxb/erv458 (PMC4682435; doi:10.1093/jxb/erv458)
Supplement: Supplementary Data [file supp_erv458_Supplementary_Tables_S1_S2_Figures_S1_S7.pdf]

KASP name and primers	Sequence
**1. *TaBradi2g14790***	
*TaBradi2g14790*_KASP1_F	gaaggtgaccaagttcatgctCCTTGTCTCCGTCCCTG
*TaBradi2g14790*_KASP1_V	gaaggtcggagtcaacggattGACAGCTCCTCCCGAG
*TaBradi2g14790*_KASP1_generic	TCGGTAATGTCTTCAGTGTTTTA
**2. *TaELF3-B1***	
*TaELF3-B1*_Kasp_F	gaaggtgaccaagttcatgctCCCTTGCAGCTCGCT
TaELF3-B1 Kasp_V	gaaggtcggagtcaacggattCCCTTGCAGCTCGCC
TaELF3-B1 Kasp2_generic	CGACCCAACACTCACG
**3. *TaELF3-D1***	
*TaELF3-B1*_ Kasp1_F	gaaggtgaccaagttcatgctTGGAGACATGACGGGAACA
*TaELF3-D1*_Kasp1_V	gaaggtcggagtcaacggattTGGAGACATGACGGGAACG
*TaELF3-D1*_Kasp1_generic	GGAAACCAGGCTTCACG
*TaELF3-D1*_ Kasp2_F	gaaggtgaccaagttcatgctGCCTCAGAATCAGTGGCTT
*TaELF3-D1*_Kasp2_V	gaaggtcggagtcaacggattGCCTCAGAATCAGTGGCTC
*TaELF3-D1*_Kasp2_generic	GTAGACGAACCCTTCCGA
**4. *TaMOT1-D1***	
*TaMOT1-D1*_KASP1_F	gaaggtgaccaagttcatgctGGCACATATAATGTAAGGATCAATCAT
*TaMOT1-D1_KASP1*_V	gaaggtcggagtcaacggattGGCACATATAATGTAAGGATGAATCAT
*TaMOT1-D1*_KASP1_generic	AATATATAAGTTAACCATCTCATGAAAGTAAG

Table S1. The KASP primer combinations used to score the same SNPs in the genes *TaBradi2g14790*, *TaELF3-B1* and *TaELF3-D1*. The molecular concentrations of the FAM (F) and VIC (V) labelled primers were 0.16mM while the generic primers were 0.4mM. The lower case in the sequence is the FAM or VIC sequence.

B. distachyon Chromosome 2 Gene number	Match with *T. aestivum* Group1	Match with *T. aestivum* Group3	Gene or marker name or EST accession number
**Bradi2g28010**	yes	no	serine/threonine-protein kinase TOR-like (BF485305)
**Bradi2g25820**	yes	no	peptide methionine sulfoxide reductase B3, chloroplastic-like
**Bradi2g19670**	yes	no	*Barley **F**lowering Locus **T3*** ([ψ]*HvFT3*)
**Bradi2g15630**	yes	no	chloroplast unusual positioning1(CHUP1) chloroplastic-like
**Bradi2g14970**	yes	no	Glucose-1-phosphate adenylyltransferase large subunit, chloroplastic/amyloplastic-like
**Bradi2g14940**	yes	no	uncharacterised
**Bradi2g14830**	yes	no	probable indole-3-acetic acid-amino synthetase GH3.5-like
**Bradi2g14790**	yes	no	*RNA polymerase sigma factor rpoD-like*
Bradi2g14780	no	partial 3B	
Bradi2g14770	Yes (**1DS**)	yes	
Bradi2g14760	no	no	
Bradi2g14750	no	partial 3B	
**Bradi2g14740**	yes	yes	
**Bradi2g14730**	yes	yes	CTP synthase-like
**Bradi2g14460**	yes	no	[α]*BJ544902*
**Bradi2g14440**	yes	3A and 3B	**Xcdo393* ([μ]*Sb09g030620*)
**Bradi2g14400**	yes	no	**v**acuolar **ATP** synthetase subunit **C** (**vatpC*) ([α]*XAL503851*)
**Bradi2g14380**	yes	no	**XCA608558*
**Bradi2g14370**	yes	no	**Xwg241*
**Bradi2g14340**	yes	no	[β]***MO**DIFIER **O**F **T**RANSCRIPTION **1** (MOT1)*
**Bradi2g14310**	yes	yes	***Ad**enylate kinase **1** (*XADK1)*
**Bradi2g14290**	yes	no	*AtELF3/Eam8/Mat-a* (early maturity)/ [α]*XBarc62*
**Bradi2g14290 3'UTR**	yes	no	[α]*XBarc62*
**Bradi2g14250**	yes	no	UDP-glucose 4-epimerase GEPI48-like (LOC100838089), mRNA
**Bradi2g14210**	yes	no	**N**ucleoside **d**iphosphate **k**inase **3** (**Ndk3*) [μ]*Sb09g030810*
**Bradi2g14190**	yes	no	uncharacterised
**Bradi2g14130**	yes	no	uncharacterised
Bradi2g14120	yes	partial	Histone deacetylase HDT2-like
Bradi2g14110	yes	yes	rho GDP-dissociation inhibitor 1-like
Bradi2g14090	partial	no	uncharacterised
Bradi2g14080	no	no	
Bradi2g14070	no	yes	
Bradi2g13870	no	yes	
Bradi2g13860	no	no	
Bradi2g13850	no	no	
Bradi2g13840	no	no	
Bradi2g13820	no	yes	
Bradi2g13810	no	yes	
Bradi2g13800	no	yes	
**Bradi2g13790**	yes	no	probable inactive leucine-rich repeat receptor-like protein kinase (At1g66830-like)
**Bradi2g13750**	yes	no	*Adaptor protein complex 3 subunit delta (AP-3)-like*

*Valarik *et al*., 2006; [α]Song *et al*., 2005; [β]Faricelli *et al*., 2010; [γ]Higgins *et al*., 2010; and [μ]Zakhrabekova *et al*., 2012; [δ]Faure *et al*., 2012; [ψ]Faure *et al*., 2007.

Table S2 The 40 syntenous *B. distachyon* genes used to define the 1DL deletion.

Wheat orthologues were assigned to chromosome arms by homology to chromosome arm sorted survey sequence as described in Materials and Methods. Of the forty genes, eleven

genes, *TaBradi2g14730*, *TaBradi2g14460*, *Bradi2g14440*, *Bradi2g14400*, *Bradi2g14380*, *Bradi2g14370*, *Bradi2g14340*, *Bradi2g14310*, *Bradi2g14290 and Bradi2g14210*, and *TaBradi2g14190* were all shown to be part of the segment that has several genes deleted from Spark, and Cadenza and they are shown in red colour (Table S2). Twelve of the forty genes had no matches with wheat group one chromosomes and these are shown in blue (Table 1). Out of the twelve genes that do not match group 1 wheat chromosomes, five matched the wheat group three chromosomes and these are *Bradi2g14070*, *Bradi2g13870*, *Bradi2g13820*, *Bradi2g13810*, and *Bradi2g13800* (Table S2).

The gene *Bradi2g14770* matched group 3 genes but the match on group 1 was on 1DS. The genes *Bradi2g14740*, *Bradi2g14120* and *Bradi2g14110*, matched homologues on both group 1 and group3 wheat chromosomes (Table S2) and these were not used to define the deletion because amplification from group 3 would not be distinguishable from group1 in the absence of polymorphism that can be used to differentiate the locations. The genes *Bradi2g14780*, *Bradi2g14750*, and *Bradi2g14440* (Table S2), matched genes on both group1 and group3 chromosomes but none of the three had sequence match with the group 3 D genome chromosome of "Chinese Spring" and hence *Bradi2g14440* was used to define the deletion. The gene *Bradi2g14730* matched both group1 and group 3 but when the genes were aligned, the group 1 genes were sufficiently different from the group 3 genes hence primers were designed to be specific to 1DL and this gene was also found to be among the deleted genes (Fig. 2). Eleven genes outside the 1DL deletion matched group 1 chromosomes only and all these were used to define the deletion (bold black Table S2).

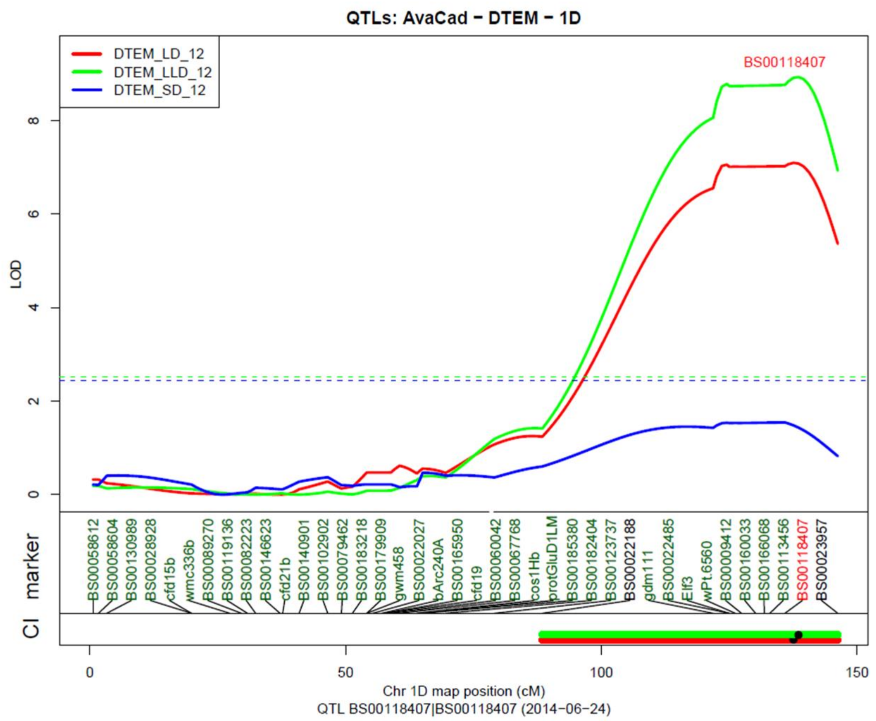

A

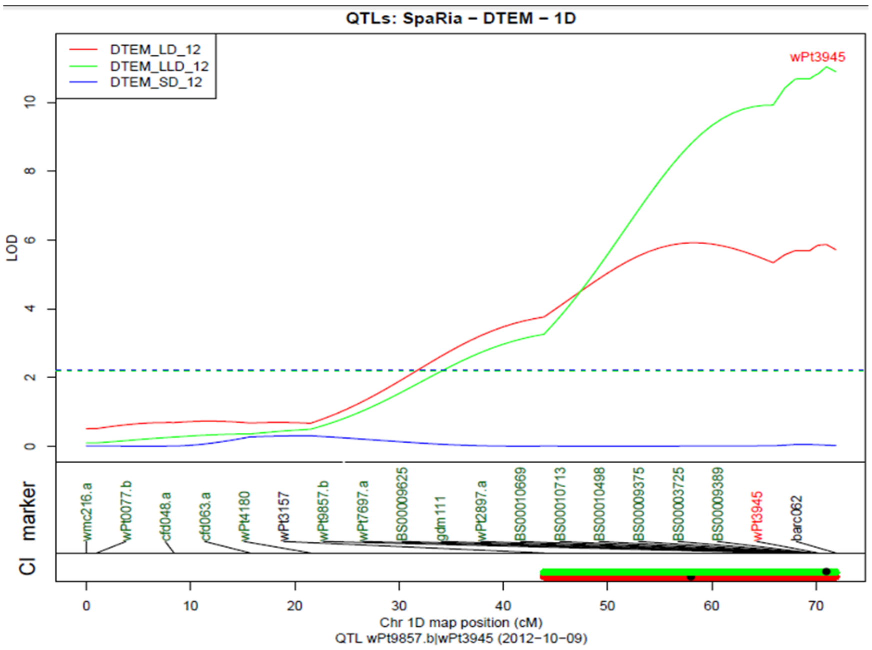

B

Fig. S1 Chromosomal location of the 1DL heading date QTL for and Spark X Rialto (A), Avalon X Cadenza (B) doubled haploid (DH) population vernalized at 7-10°C for 8 weeks and grown in short days (SD) 10 hrs light, long days (LD) 16 hrs light and very long days (LLD) 20 hrs light. The Avalon X cadenza (B) shows *TaELF3-D1* coinciding with the peak of the QTL (B).

```
1BL_TaELF3        CCGGGTGGTCCCGCACACGGCGCGCACCGCGTCGGAGTCGGCGGCGCGCATCTTCCGGTC 60
1AL_TaELF3        CCGGGTGGTCCCGCACACAGCGCGCACCGCGTCAGAGTCGGCGGCGCGCATCTTCCGGTC 60
TuELF3            CCGGGTGGTCCCGCACACAGCGCGCACCGCGTCAGAGTCGGCGGCGCGCATCTTCCGGTC 60
AtELF3            CCGGGTGGTACCGCACACGGCGCGCACCGCGTCGGAGTCGGCAGCGCGCATCTTCCGGTC 60
1DL_TaELF3        CCGGGTGGTACCGCACACGGCGCGCACCGCGTCGGAGTCGGCAGCGCGCATCTTCCGGTC 60
XBarc62           ------------------------------------------------------------
                                                      Stop!
1BL_TaELF3        GATCCAGATGGAGAGGCAGCAGAACGGCCCGTGAccgagcgaccgcatgcggtgcttggc 120
1AL_TaELF3        GATCCAGATGGAGAGGCAGCAGAACGGCCCGTGAccgagcgaccgcatgcagtggttggc 120
TuELF3            GATCCAGATGGAGAGGCAGCAGAACGGCCCGTGAccgagcgaccgcatgcggtggttggc 120
AtELF3            GATCCAGATGGAGAGGCAGCAGAACGGCCCGTGAcagagcgaccgcatgcggtggttggc 120
1DL_TaELF3        GATCCAGATGGAGAGGCAGCAGAACGGCCCGTGAcagagcgaccgcatgcggtggttggc 120
XBarc62           ------------------------------------------------------------

1BL_TaELF3        cggcacaaagaaa---aggaaggaagaaagcttagccaattagcgcttgggagtatgttg 177
1AL_TaELF3        cggcacaaagaaagaaaggaaggaagaaagcttagccaattagcgcttgggagtatgttg 180
TuELF3            cggcacaaagaaagaaaggaaggaagaaagcttagccaattagcgcttgggagtatgttg 180
AtELF3            cggcacaaagaaagaaaggaaggaagaaagcttagccaattagagcttgggagtatgttg 180
1DL_TaELF3        cggcacaaagaaagaaaggaaggaagaaagcttagccaattagagcttgggagtatgttg 180
XBarc62           ------------------------------------caattagagcttgggagtatgttg 24
                                      Primer 1            ******* ****************
1BL_TaELF3        ttattttgagcaggca----------------atagcattcctatatat--ttgtccct 219
1AL_TaELF3        ttattttgagcaggcaggcag----caagcaaatagcattcctatatat--ttgtccct 234
TuELF3            ttattttgagcaggcaggcag----caagcaaatagcattcctatatat--ttgtccct 234
AtELF3            ttattttgagcaggcaggcaggcagcaagcaaatagcattcctatatatatttgtccct 240
1DL_TaELF3        ttattttgagcaggcaggcaggcagcaagcaaatagcattcctatatatatttgtccct 240
XBarc62           ttattttgagcaggcaggcaggcagcaagcaaatagcattcctatatatatttgtccct 84
                  *****************                *****************  ********

1BL_TaELF3        cggcgtcggtaaaagcgaattttggcgactgtaaatatgcaaagcaaagcaggacttg- 278
1AL_TaELF3        cggtgtcggtaaaagcgaattttggcgactgtaaatatgcaaagcaaagcaggactt-- 294
TuELF3            cggtgtcggtaaaagcgaattttggcgactgtaaatatgcaaagcaaagcaggactt-- 294
AtELF3            ctgcgtcggtaaaagcgaattttggcgactgtaaatatgcaaagcaaagcaggacttgt 300
1DL_TaELF3        ctgcgtcggtaaaagcgaattttggcgactgtaaatatgcaaagcaaagcaggacttgt 300
XBarc62           ctgcgtcggtaaaagcgaattttggcgactgtaaatatgcaaagcaaagcaggacttgt 144
                  * * *******************************************************
1BL_TaELF3        ------------------------------------------------tatctatctatct 291
1AL_TaELF3        ------gccagaacagaatgagtgctggttgggcaaagcaggacttgtatctatcaatct 346
TuELF3            ------gccagaacagaatgagtgctggttgggcaaagcaggacttgtatctatcaatct 346
AtELF3            atgaaggccagaacagaatgagtgctggttgggcaaagcaggacttgtatctatctatct 360
1DL_TaELF3        atgaaggccagaacagaatgagtgctggttgggcaaagcaggacttgtatctatctatct 360
XBarc62           atgaaggccagaacagaatgagtgctggttgggcaaagcaggacttgtatctatctatct 204
                Primer2         Primer3                                  ******** ****
                                                               XBarc62 SSR start

1BL_TaELF3        atttatctat--------------------ctatgccacaatgaaaactgtaactgttagt 332
1AL_TaELF3        acctatctat--------------------ttatgccagaatgaaaactgtaactgttagt 387
TuELF3            atctatctat--------------------ttatgccagaatgaaaactgtaactgttagt 387
AtELF3            atctatctatctatccatctatctatctactatgccagaatgaaaactgtaactgttagt 420
1DL_TaELF3        atctatctatctatctatct--------actatgccagaatgaaaactgtaactgttagt 412
XBarc62           atctatctatctatctatct--------actatgccagaatgaaaactgcaactgttagt 256
                  *  ******* ***                    **** *********** **********

                      XBarc62 SSR end
1BL_TaELF3        tttaggtgtatgt----ctcaggcaaaaagaagtgaaacctgggagtgatgtctc-tgct 387
1AL_TaELF3        gttaggtgtatgtatgtctcaggcaaaaagaagtgaaacctgggagtgatggctc-tgct 446
TuELF3            gttaggtgtatgtatgtctcaggcaaaaagaagtgaaacctgggagtgatggctc-tgct 446
AtELF3            gttaggtgtatgtatgtctcaggcaaaaagaagtgaaacctgggagtgatggctc-tgct 479
1DL_TaELF3        gttaggtgtatgtatgtctcaggcaaaaagaagtgaaacctgggagtgatggctc-tgct 471
XBarc62           gttaggtgtatgtatgtctcaggcaaaaagaagtgaaacctgggagtgatggctcgtgct 316
                    ************    ********************************** *** ****
                      Primer 4
1BL_TaELF3        gtattgtctatgtatgtgcagctatattgcctgctgatatcttat--------------- 432
1AL_TaELF3        atattatctatgtatgtgcagctatagtgcctgctgatatcttat--------------- 491
TuELF3            atattatctatgtatgtgcagctatagtgcctgctgatatcttat--------------- 491
AtELF3            gtattatctatgtatgtccagctatattgcctgctgatatcttatgggcctctttgattc 539
1DL_TaELF3        gtattatctatgtatgtccagctatattgcctgctgatatcttatgggcctctttgattc 531
XBarc62           gtattatctatgtatgtccagctatattgcct---------------------------- 348
                   **** *********** ******** *****
```

Fig. S2 Position of the *Xbarc62* simple sequence repeat (SSR) 194 bases from the stop codon in the 3’ untranslated region (UTR) of *TaELF3* gene. The letters in capital are part of the fourth exon of the *TaELF3* gene and the octagon written stop marks the position of the stop

codon (TGA). The sequence labelled *Xbarc62* is the expressed sequence tag (EST) accession BV211449 used to design *the XBarc 62* SSR marker (Song *et al*., 2005). The PCR primers are underlined and labelled primer 1, Primer 2 (designed to be specific to 1DL) while primer 3 and 4 were designed by Song *et al*., (2005) and amplify from both 1DL and 1AL (Fig. 1). The difference between primer 2 and 3 is that primer 3 is shown by the single underline while primer 2 includes the whole of primer 3 and four additional bases (gaag) shown by double underlining which make primer 2 1DL specific (fig1). The D homeologue is 11bp longer than the A and B homeologues in the region between the two black downward arrows (Fig. 1). The start and end of the ‘ATCT’ SSR that is scored by the *XBac62* marker is shown by the upward arrows and also by the dotted underline and the black horizontal bar flanked by the two upward facing arrows.

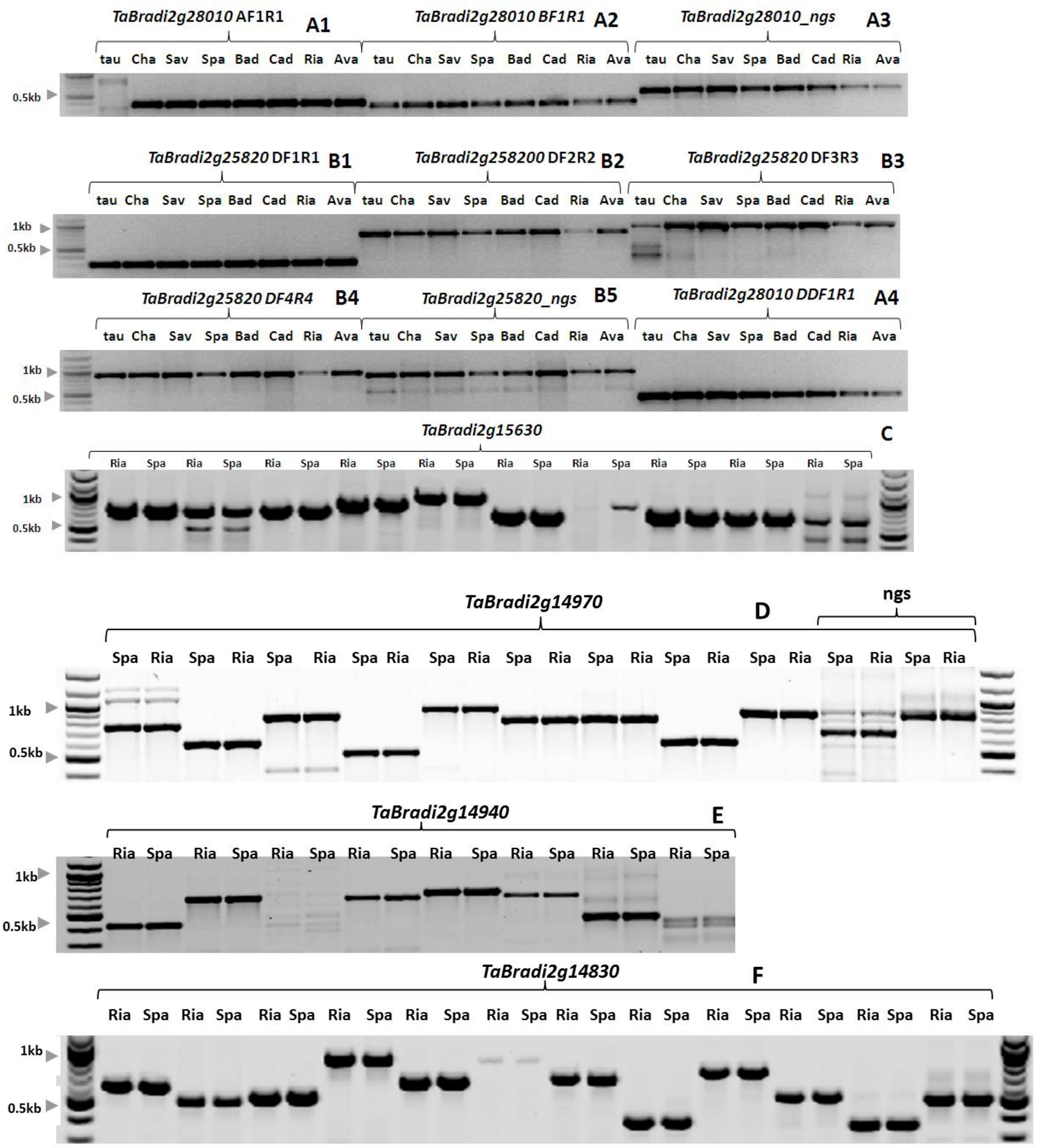

Fig. S3.1 The 1D genes that match the *B. distachyon* chromosome 2 genes that were used to define the proximal end of the 1DL deletion. Non genome specific primers are shown as ngs. Key: tau = *A. tauschii*, Cha = Charger, Sav = Savannah, Bad = Badger, Cad = Cadenza, Ria= Rialto, Ava = Avalon.

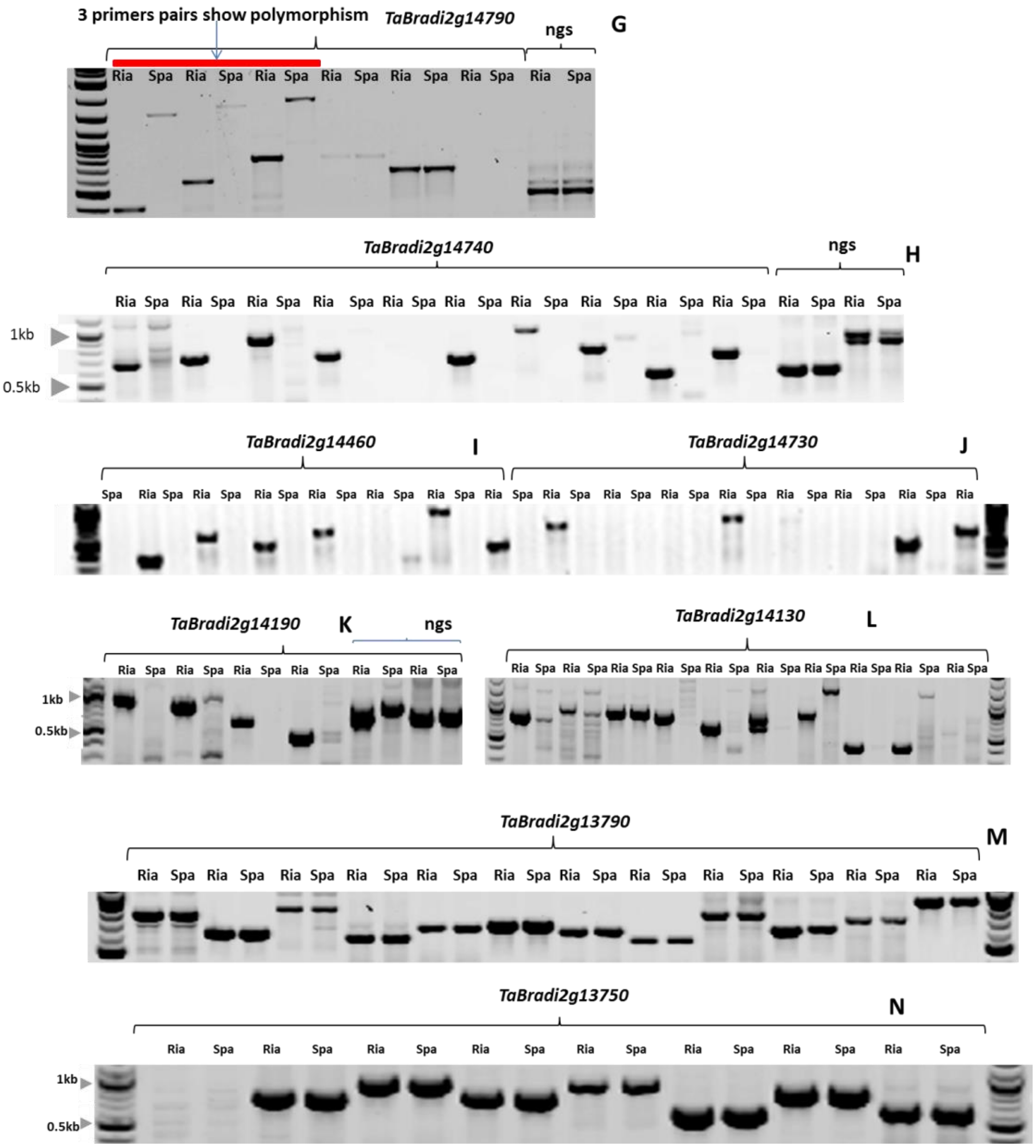

Fig. S3.2 The wheat chromosome 1DL genes that match the *B. distachyon* chromosome 2 genes that were used to show that the 1DL deletion includes *TaBradi2g14740* (H), *TaBradi2g14460* (I), *TaBradi2g14730* (J), *TaBradi2g14190* (K) and *TaBradi2g14130* (L). Key: Spa = Spark, Ria = Rialto. The genes *TaBradi2g1379*0 and *TaBradi2g13750* define the 1DL deletion on the distal end of the deletion. The gene *TaBradi2g14130* (L) amplifies on the 5' end for both Spark and Rialto and about 1Kb of this gene was sequenced from both varieties but the rest of the gene is not amplified from Spark suggesting that the distal deletion breakpoint maybe within this gene.

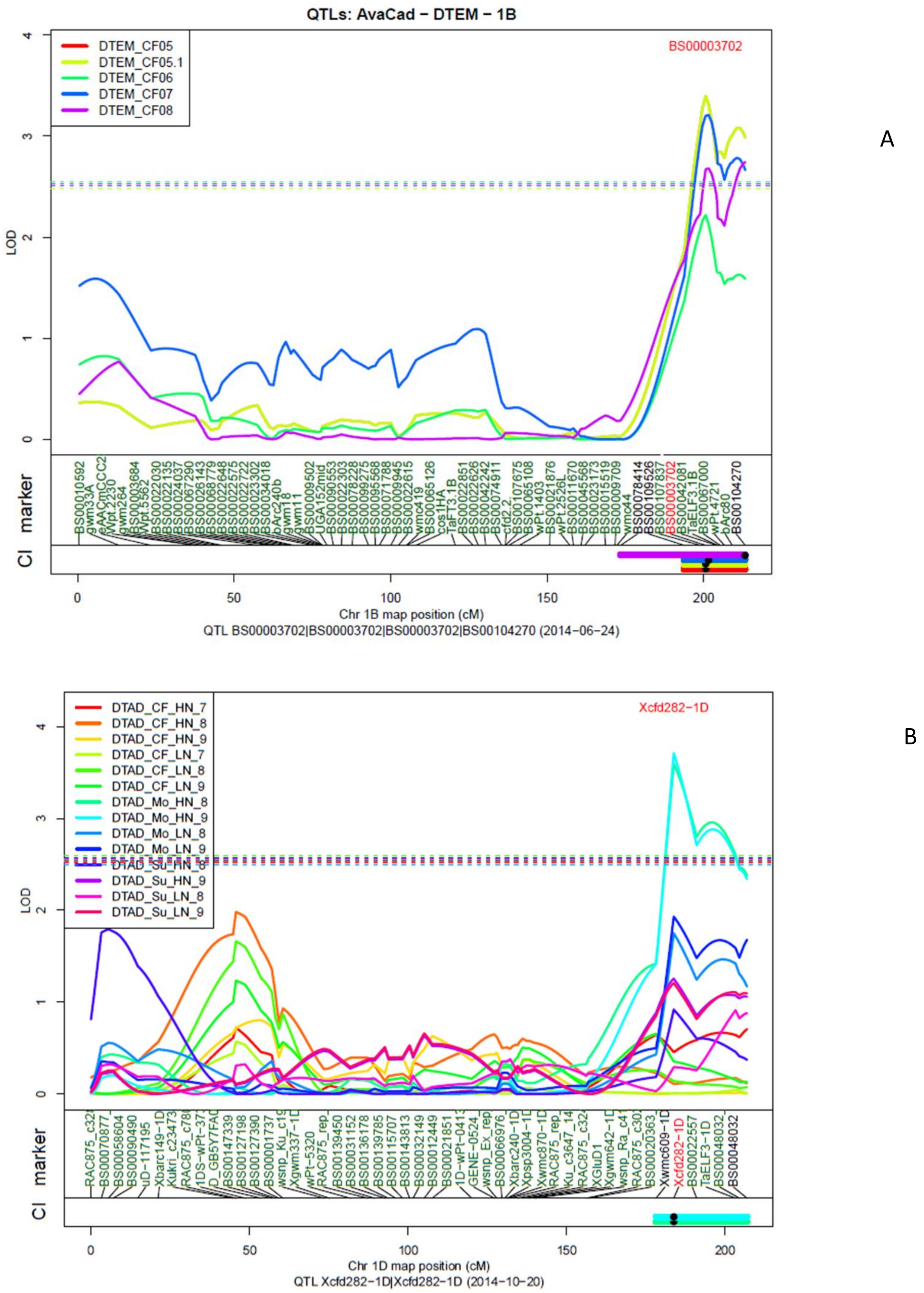

Fig. S4 Chromosomal location of the 1BL heading QTL for and Avalon X Cadenza (A), and 1DL heading date QTL for Savannah X Rialto (B) doubled haploid (DH) population grown in the field. Both QTLs show that *TaELF3-B1* and *TaELF3-D1* coincide with the peak of the QTLs. The QTLs seem to respond to environmental cues given that they are not significant in some years.

Recombinant class	Part of 1DL				1DL deletion			Number of recombinants and flowering time
	TaBradi2g19670	Xcfd63	Xgdm111	TaBradi2g14790	TaBradi2g14740	XBarc62	TaBradi2g14190	
Outliers								
P1_78	b	b	b	b	b	b	b	early
P2_23/P3_65	a	a	a	a	a	a	a	late
P2_92	a	a	b					late
P2_39/83	a	a	b	b	b	b	b	early
P2_64	a	a	a/b	b	b	b	b	early
P1_10/92/P3_60/88	b	b	a/b	b	b	b	b	early
P1_2/32/46/88	b	b	a	a	a	a	a	late
P3_17/26/P4_72/86	b	b	a	a	a	a	a	late
P3_48/60	a	a	b	b	b	b	b	early
P3_2	a	a	a/b	b	a	a	a	late
P3_35/P4_95	a	a	b	b	a	a	a	late
P4_21	b	b	a/b	b	b	b	b	early
P4_85	b	a/b	b	b	b	b	b	early

Figure S5. The genotypes of the outliers in the *Eps-D1* region. P1_78 has no recombination as well as P2_23 and P3_65 suggesting that there may be another gene(s) in the background responsible for the phenotype of the outliers.

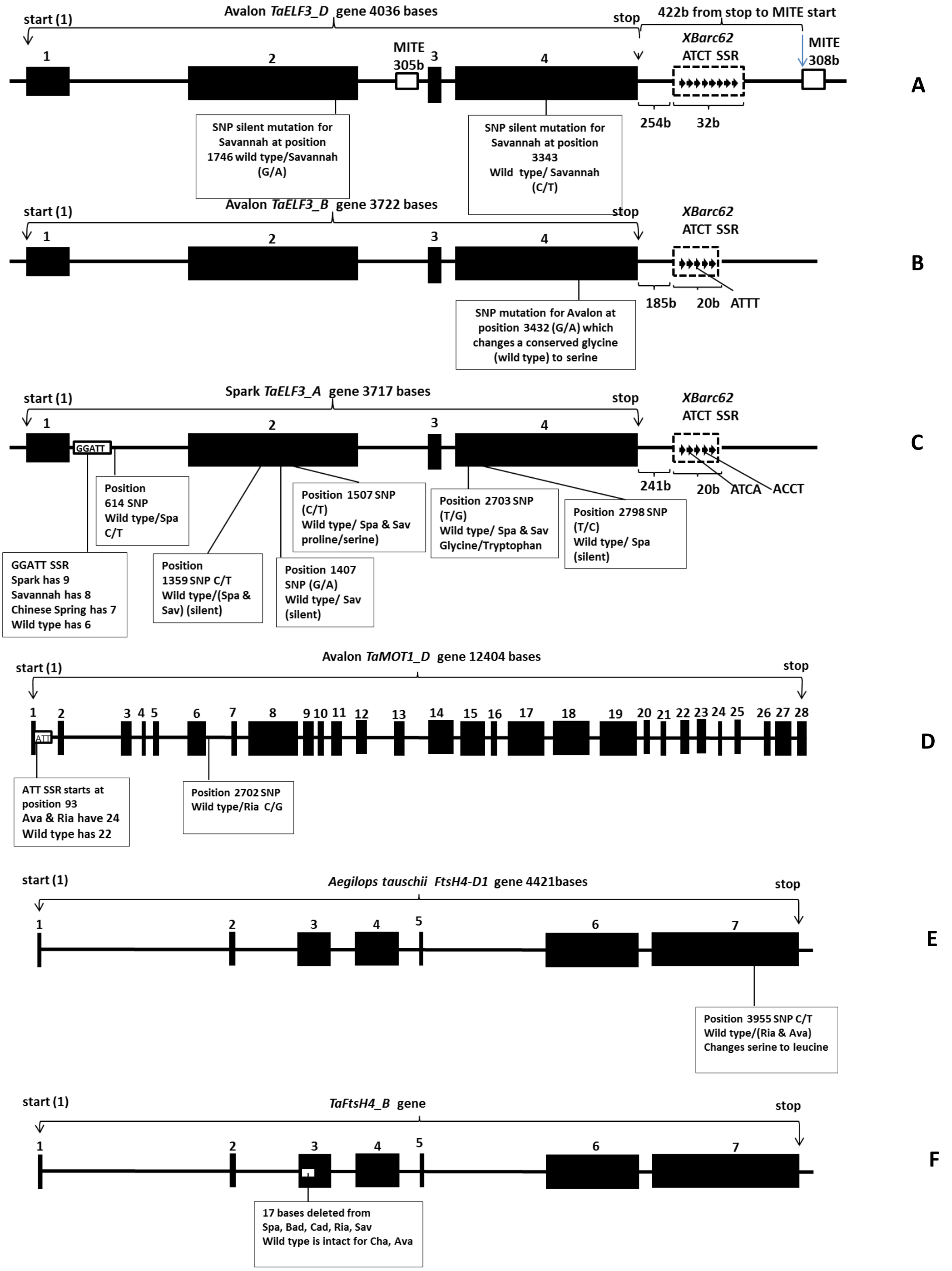

Figure S6 Mutations at the *TaELF3-D1* (A), *TaELF3-B1* (B), *TaELF3-A1* (C) *TaMOT1-D1* (D), *TaFTSH4-D1* (E), and *TaFTSH4-B1* (F) genes. The black rectangles with numbers at the top are the exons (4 for *TaELF3* genes (A, B and C), 28 for *TaMOT1-D1* (D) and 7 for *TaFTSH4* genes (E and F)). The unshaded solid rectangles are the miniature inverted

transposable elements (MITE) in intron 2 and the 3’UTR of *TaELF3-D1* gene 305 and 308 bases long respectively (A). The dotted rectangles for *TaELF3-D1*, *TaELF3-B1* and *TaELF3-A1* are the *XBarc62* ATCT repeats and each arrow in the dotted box represents a single ATCT repeat of which the third one for *TaELF3-B1* is ATTT and the second and fourth for *TaELF3-A1* are ATCA and ACCT respectively. The *XBarc62* ATCT SSR has 8 repeats for the D copy and 5 each for the A and B copies of *TaELF3*. Key: Rialto (Ria), Spark (Spa), Avalon (Ava), Cadenza (Cad), Charger (Cha), Badger (Bad) and Savannah (Sav)

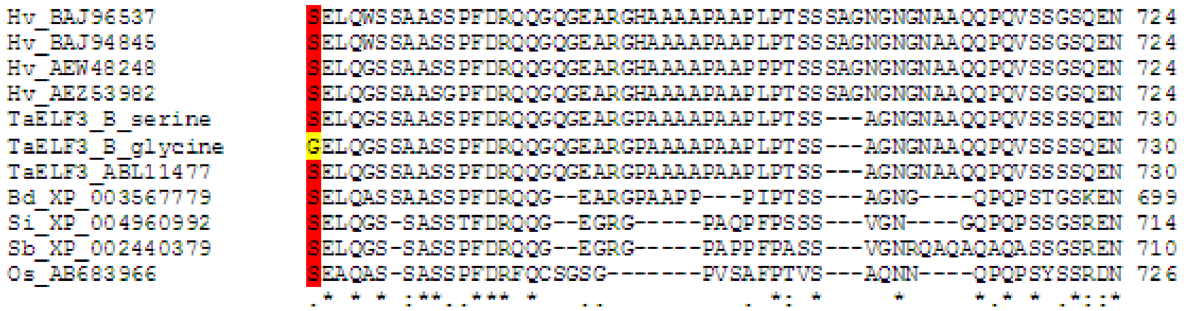

Fig. S7 The partial alignment of *ELF3* genes from *Hordeum vulgare* (*Hv*), *Triticum aestivum* (*Ta*), *Brachypodium distachyon* (*Bd*), *Sorghum bicolor* (*Sb*), Si and *Oryza sativa* (*Os*). The alignment shows the change in the conserved serine (S) to glycine (G). This mutation distinguishes Avalon (mutant) from Cadenza (wild type).
